# Supplementary material for: Adverse or therapeutic? A mixed-methods study investigating adverse effects of Mindfulness-Based Cognitive Therapy in bipolar disorder
Source: PLoS One. 2021 Nov 4;16(11):e0259167. doi: 10.1371/journal.pone.0259167 (PMC8568103; doi:10.1371/journal.pone.0259167)
Supplement: S1 Table — (DOCX) [file pone.0259167.s001.docx]

| **S1 Table: Self-report questionnaire to monitor the occurrence of adverse effects (AEs) during MBCT** | | | | |
| --- | --- | --- | --- | --- |
|  | During the week | At the end of the week | | |
| **Adverse or unexpected effect during mindfulness exercises***  *formal exercises, such as bodyscan, sitting meditation or mindful moving exercises | **Present**  Count when present  E.g.: \| \| \| | **Relation with MBCT**  “Yes / No / Maybe” | **Intensity**  Scale 1 – 10  1: none  10: a lot | **Action**  1: No action  2: Paused  3: Stopped  4: Asked for help |
| 1. Re-experiencing of traumatic memories (as if you are experiencing it again) |  |  |  |  |
| 1. Overwhelming feelings of depression, dysphoria, or grief |  |  |  |  |
| 1. Uncontrollable feelings of happiness or mania |  |  |  |  |
| 1. Uncontrollable feelings of agitation of irritability |  |  |  |  |
| 1. Sudden (increase of) feelings of anxiety or panic |  |  |  |  |
| 1. Strange or unreal feelings towards your surroundings or the world, as if you are not part of it (derealization) |  |  |  |  |
| 1. Strange or unreal feelings towards yourself (depersonalization) |  |  |  |  |
| 1. Distrust towards others |  |  |  |  |
| 1. Doubting yourself |  |  |  |  |
| 1. Seeing things that other people do not see (visual hallucinations) |  |  |  |  |
| 1. Hearing things that other people do not hear (auditive hallucinations) |  |  |  |  |
| 1. Specific bodily sensations, namely………………………………………………………   …………………………………………………………………………  ………………………………………………………………………… |  |  |  |  |
| Other adverse or unexpected effects, namely..  ………………………………………………………………………..  …………………………………………………………………………  …………………………………………………………………………  …………………………………………………………………………  ………………………………………………………………………… | ………………..  ………………..  ………………..  ………………..  ……………….. | ……………  ……………  ……………  ……………  …………… | ………………  ………………  ………………  ………………  ……………… | ………………..  ………………..  ………………..  ………………..  ……………….. |
| Number of times practicing mindfulness exercises this week: ……………………… | | | | |
| Room for remarks: | | | | |
