# Supplementary material for: Adverse or therapeutic? A mixed-methods study investigating adverse effects of Mindfulness-Based Cognitive Therapy in bipolar disorder
Source: PLoS One. 2021 Nov 4;16(11):e0259167. doi: 10.1371/journal.pone.0259167 (PMC8568103; doi:10.1371/journal.pone.0259167)
Supplement: S2 Table — (DOCX) [file pone.0259167.s002.docx]

| **S2 Table: Topic-list used during interviews**  **1. Meditation-related adverse effects (AEs)**   - *What kind of adverse or unexpected experience(s) did you have that you considered to be related to MBCT?* - *Did this experience have an impact on your daily life?*   **2. Responses to AEs**   - *How did you or others respond to these experiences?* - *Which actions or strategies have you found particularly helpful / unhelpful?*   **3. Interpretation of AEs**   - *To what extent do you think these experiences were caused by MBCT?* - *What other factors might have had an influence on the occurrence of these experiences?* - *When looking back, do you think these experiences were only negative, or might they be part of some kind of growth or development?* |
| --- |
