# Supplementary material for: Adverse or therapeutic? A mixed-methods study investigating adverse effects of Mindfulness-Based Cognitive Therapy in bipolar disorder
Source: PLoS One. 2021 Nov 4;16(11):e0259167. doi: 10.1371/journal.pone.0259167 (PMC8568103; doi:10.1371/journal.pone.0259167)
Supplement: S3 Table — (DOCX) [file pone.0259167.s003.docx]

| **S3 Table. Phenomenology Codebook: Number and proportions of predetermined (Lindahl et al., 2017) found adverse events in interviews during Mindfulness-Based Cognitive Therapy in patients with bipolar disorder (*n* = 19)** | |
| --- | --- |
| **Domains and codes** | **Frequency *n*** |
| Affective domain | |
| Fear, Anxiety, Panic, or Paranoia | 16 |
| Depression, Dysphoria, or Grief | 13 |
| Change in Doubt, Faith, Trust, or Commitment | 9 |
| Agitation or Irritability | 8 |
| Re-experiencing of Traumatic Memories or Affect without Recollection | 7 |
| Rage, Anger, or Aggression | 5 |
| Positive Affect | 4 |
| Crying or Laughing | 3 |
| Self-Conscious Emotions | 2 |
| Affective Flattening, Emotional Detachment, or Alexithymia | 1 |
| Affective Liability | 1 |
| Empathic or Affiliative Changes | 1 |
| Suicidality | 0 |
| Cognitive domain | |
| Increased Cognitive Processing | 3 |
| Clarity | 2 |
| Change in Worldview | 1 |
| Change in Executive Functioning | 0 |
| Delusional, Irrational, or Paranormal Beliefs | 0 |
| Disintegration of Conceptual Meaning Structures | 0 |
| Mental Stillness | 0 |
| Meta-Cognition | 0 |
| Scrupulosity | 0 |
| Vivid Imagery | 0 |
| Conative Domain | |
| Change in Motivation or Goal | 2 |
| Change in Effort or Striving | 1 |
| Anhedonia or Avolition | 0 |
| Perceptual Domain | |
| Derealization | 3 |
| Hallucinations, Visions, or Illusions | 2 |
| Somatosensory Changes | 2 |
| Distortions in Time or Space | 1 |
| Dissolution of Objects or Phenomena | 0 |
| Perceptual Hypersensitivity | 0 |
| Visual Lights | 0 |
| Sense of Self Domain | |
| Change in Sense of Embodiment | 3 |
| Change in Self-Other or Self-World Boundaries | 2 |
| Loss of Sense of Ownership | 2 |
| Change in Narrative Self | 1 |
| Loss of Sense of Agency | 0 |
| Loss of Sense of Basic Self | 0 |
| Social Domain | |
| Increased Sociality | 2 |
| Change in Relationship to Meditation Community | 1 |
| Occupational Impairment | 1 |
| Social Impairment | 1 |
| Integration Following Retreat or Intensive Practice | 0 |
| Somatic Domain | |
| Breathing Changes | 6 |
| Gastrointestinal Distress or Nausea | 5 |
| Pressure, Tension, or Release of Pressure, Tension | 5 |
| Somatic Energy | 5 |
| Sleep Changes | 4 |
| Thermal Changes | 4 |
| Cardiac Changes | 3 |
| Fatigue or Weakness | 3 |
| Dizziness or Syncope | 2 |
| Parasomnias | 2 |
| Headaches or Head Pressure | 1 |
| Appetitive or Weight Changes | 0 |
| Involuntary Movements | 0 |
| Pain | 0 |
| Sexuality-Related Changes | 0 |
