# Supplementary material for: Adverse or therapeutic? A mixed-methods study investigating adverse effects of Mindfulness-Based Cognitive Therapy in bipolar disorder
Source: PLoS One. 2021 Nov 4;16(11):e0259167. doi: 10.1371/journal.pone.0259167 (PMC8568103; doi:10.1371/journal.pone.0259167)
Supplement: S4 Table — (DOCX) [file pone.0259167.s004.docx]

| **S4 Table. Influencing Factors Codebook: Percentage of predetermined (Lindahl et al. 2017) and newly found influencing factors of adverse effects as reported by patients with bipolar disorder during Mindfulness-Based Cognitive Therapy (*n* = 19), illustrated with quotes** | |
| --- | --- |
| **Domains and codes (*n*)** | **Corresponding quotes** |
| Predisposing factors | |
| Psychiatric history (19) | I kind of relived the psychosis I had. (#7) |
| *Current mood symptoms of BD (8)* | I notice that the more depressed I am, the more susceptible I become to panic attacks (AE). (#9) |
| Trauma history (7) | Between the ages of ten and fifteen, a lot of things happened that were traumatic for me, and so those then surfaced again strongly. (#3) |
| Personality or temperament (6) | I'm too much of a perfectionist. I wanted to do things as well as I could and that caused more anxiety. (#8) |
| Medical history (3) | I have a genetic disorder, a skin disease... and I was in so much pain during the fourth or fifth session... sitting was almost impossible. (#14) |
| Early life relationships (3) | But it’s happened to my grandmother on various occasions, and I take after her a lot and she also has the same diagnosis and experienced this as well (peripheral symptoms of psychosis) ... It scares me a bit. (#19) |
| Relationship beyond meditation community (2) | No, I really think it was a combination of an incident that occurred just before the course started, someone harassed me... and I think that actually brought things to the surface, and affected me during the course. (#10) |
| *History with AE (3)* | And years ago, ten or twenty years ago now, I’d wanted to do yoga. It went wrong then too... The mere mention of the word breathing.... (#2) |
| Intention, motivation, or goals (5) | I thought well this will be nice and relaxing and I’m going to enjoy this (mindfulness training), but I didn't expect it would also be challenging. (#4) |
| Precipitating factors | |
| Type of practice |  |
| *Bodyscan (9)* | It’s especially related to the body scan we’ve been practicing... the pace is way too slow for me, and I get incredibly annoyed by that.... (#11) |
| *Sitting meditation (6)* | And that was especially during the sitting meditation. And somewhere I did know it really was about me, but it just seemed like it was someone else.... (#9) |
| *Focus on breathing (4)* | Just thinking about breathing, or even just talking about that breathing makes me feel anxious. (#2) |
| *Walking meditation (4)* | When I was doing walking meditation, old (traumatic) thoughts would still sometimes pop up. (#9) |
| *Standing movement exercises (3)* | I got very dizzy during the physical exercises... (#17) |
| *Automatic manic thought questionnaire (2)* | I think you had to name some manic things about yourself ...how you feel and stuff like that. And that's pretty intense... So a lot of things come back then. And some of that feels like you’re reliving it. (#6) |
| Amount of practice (5) | It was really during the long meditation...Then they (traumatic memories) would resurface. (#10) |
| Surroundings (8) | Yeah one distracting factor was that there were often noises next door or outside the building. That annoyed me, and then I couldn't concentrate well. (#11) |
| Relationship to teacher (5) | The way she (the trainer) talked, the intensity of how she did that, her voice, her intonation, the exact way she conveyed it... took me back to a core (inner vulnerability) where I didn't want to be. (#7) |
| Relationship within meditation community (3) | And the stories from other fellow course participants, I think that triggered something as well.... (#15) |
| Relationship beyond meditation community (3) | This whole course coincided with a period when my mother was in a bad way and also eventually passed away. (#1) |
| Perpetuating factors | |
| Practice approach |  |
| *Counting (1)* | We were told you could also count your breathing, that it might help more. Not in and out, but one, two, one, one. So then I tried that again, but that only made it worse. (#2) |
| (Automatic) Coping strategies (4) | I also have this fear of failure or something. So I thought I have to do this right, because I'm doing a course. And because of that, I really started to doubt myself and became insecure. (#5) |
| Amount of practice (1) | So if I continue practicing meditation, I thought that it (the traumatic reliving of events) might just melt away and fade away again, but that wasn’t the case. (#3) |
| Surroundings (1) | What I do notice then is that I get distracted by someone else.... I'll be sitting there and I’ll hear that and I just can't be okay with it... that's frustrating. (#5) |
| Relationship to teacher (3) | The trainer was actually... every time we had another session, she never ask anything like how did you experience things (AEs during home practice) in the past week. (#13) |
| Relationship within meditation community (5) | But I didn't want the group to see that, and because of that it kept getting worse. (#4) |
| Psychotherapy or medical treatment (1) | So I went to my GP. It really was some kind of panic attack. GP wanted to prescribe oxazepam. But I'm already on a lot of medication. So yeah... that didn't help either. (#2) |
| Mitigating factors | |
|  |  |
| Practice approach |  |
| *Focus on breathing (7)* | But I do notice that nowadays when the (traumatic) feeling surfaces, when in the moment I can’t fully cope with the feeling or have a hard time experiencing it, that I simply move straight on to breathing. (#12) |
| *Inquiry (1)* | What helped most was what I myself said. That I should put my own thoughts and feelings into words. That was then useful to me. (#18) |
| *Stopping practice (7)* | The moment those (traumatic) images surface I have to stop and do something else for a while. Take a break for a quick cup of tea and then go back to it. (#10) |
| Mindfulness skills |  |
| *Grounding Activity (15)* | Yeah... actually just touching something for a moment.... Something... that like brings you back to the now. (#1) |
| *Allowing / accepting (7)* | Yeah, but that I also allow myself to feel emotions. And that I also let them be. Because before I was always like I can't cry, crying was for wimps. And I did notice that the more I worked on that, the more I began to accept that in myself. (#9) |
| *Decentering (6)* | I was actually just trying to observe it: just observe it and you'll see that you don't have to be afraid of it. (#5) |
| *Soothing (8)* | That you psych yourself up to say what room you're in and what you're doing at that moment, that it's not scary and it's not dangerous, that nothing's going to happen now, that you're no different than you were 15 minutes ago… that you're actually looking at yourself with kindness or compassion. (#8) |
| Amount of practice (10) | I never do my exercises for longer than 20 minutes now. (#8) |
| Relationship to teacher (10) | That if, for example, I said: gosh, I didn't actually like that at all, and it didn't feel good at all. That it was fine and I was allowed to feel that way. (#9) |
| Relationship within meditation community (9) | Well in any case nice to hear that others can relate to it and that you’re not alone. (#4) |
| Relationship beyond meditation community (7) | He (partner) simply offered a listening ear.... Was a pillar of support.... purely being able to talk, without it leading to a judgment or solution, but just that. (#15) |
| Medication (3) | About 4 or 5 times during the course I had to take my medication (oxazepam 10 mg), because my mind and body were so strung out. (#9) |
| Psychotherapy or medical treatment (3) | I raised the alarm right away and I spoke to my therapist. When I'm done with mindfulness training I’ll go back to EMDR after all.... (#10) |
| **Consequences domain** | |
| *Course over time* |  |
| *AE decrease over time (10)* | No, after that, it eased (clarity and fear of developing mood episodes), so it really balanced out well. (#16) |
| *AE less impact over time (4)* | At first, it (depersonalization) caused me anxiety. And later ... well ... it simply didn't anymore, because I recognised the feeling. No longer so threatening, even though it was still there. (#1) |
| *AE increase / no change over time (6)* | Yeah, it wasn't like that right away, it really increased (breathing changes and panic). It kept getting worse. (#2) |
| *Interpretation AE* |  |
| *AE part of growth process (11)* | But with what I know now, that it worked as a magnifying glass, but also gave me some very good tools. I don't know if I could have reached this point without going through that (AEs). (#19) |
| *AE not part of growth process (8)* | I can't see anything in it that helped me (panic).... purely negative experience. (#2) |
| *Note:* Categories and codes presented in *Italics* are newly found codes in the Influencing Factors Codebook. | |
