# Supplementary material for: Adverse or therapeutic? A mixed-methods study investigating adverse effects of Mindfulness-Based Cognitive Therapy in bipolar disorder
Source: PLoS One. 2021 Nov 4;16(11):e0259167. doi: 10.1371/journal.pone.0259167 (PMC8568103; doi:10.1371/journal.pone.0259167)
Supplement: Attachment — Submitted filename: Review - PONE-D-21-18959.docx [file pone.0259167.s007.docx]

Thank you for the opportunity to review this manuscript of a well-designed study examining AEs in an impressive sample of patients diagnosed with bipolar disorder undergoing MBCT. There is much to recommend this article, which emerges at a time when there is growing interest in AEs in mindfulness based programs. The monitoring of adverse events was well thought out and well executed. With some important revisions pertaining to interpretation, clarity, and description of the findings, I believe this would make a valuable addition to the literature on AEs in mindfulness programs. Below are some general comments, followed by specific items to address.

As I have mentioned, certain components of this manuscript would benefit from revision. Features of the methodology would benefit from more detailed explanation, and aspects of the study methods suggest (mostly minor) revisions to the authors’ interpretations of its findings. Notably, the qualitative analysis framework should be explained in greater detail, including the specific framework used and the steps employed in that framework. Elements of the qualitative data analysis and categorization were unclear to this reviewer, including the designation of SAEs vs. AEs, how the relevant findings emerged. Some examples: were *mitigating factors* queried specifically, or spontaneously volunteered by the interviewers? Were the categories etic or emic? Similarly, how were some of the predisposing factors determined? If psychiatric history was only gleaned from spontaneous responses during the interview—the interview, as currently described, only lists 3 topics which do not include psychiatric history—then estimates of psychiatric are likely to be inaccurate. Was this information gathered in any other way? If not, this should be stated clearly, and listed as a limitation of the study and its interpretation. It was also not clear why those who had dropped were excluded from consideration as individuals who may have had potential SAEs. For instance, although this is listed briefly as a limitation in the study, it seems that if a suicide occurred for one of the patients, it would be premature to foreclose the possibility of it being an AE in the study. A number of similar concerns are listed point by point below. A broader methodological concern is that the authors describe this research as “confirming” Lindahl et al.’s (2017) categories, but the study does not seem optimally designed to confirm, rather than to observe or apply, Lindahl, et al.’s framework (e.g., given the smaller number of people reporting AEs in the current study, and the difference between Buddhist meditators and MBCT patients). This does not detract from the merit of the study, but does make this reviewer hesitant about interpreting this research as “confirmatory.”

This paper should also enter into closer conversation with some recent literature. Namely, Britton, et al.’s (2021) paper was likely published after submission of the present manuscript, but provides valuable guidelines for measuring harms, severity, duration, etc. Another recent paper that the authors may not have encountered at the time of submission is Goldberg, et al. (2021), which also addresses the prevalence of adverse experiences in mindfulness. Other research that went unmentioned but is probably relevant is Schlosser, et al. (Schlosser et al., 2019). Just as the Lindahl, et al. paper provided a useful typology of meditation-related challenges, the recent Britton, et al. (2021) paper provides guidelines (which the authors do not have to follow, but should be aware of/in dialogue with) for measuring harms in MBPs. It is to the current authors’ credit that they have done a very rigorous job in measuring harms, so this should not present much of a challenge. Another question related to the background literature pertains to the role of Lindahl, et al.’s work in this manuscript. The authors do impressive work applying the domains observed by Lindahl, et al., but should also mention early on that the preceding work was done in long-term Western Buddhist meditators, and generalization to MBIs should be undertaken with caution.

**Abstract**

- “Interviews were analysed with directed content analysis, using an existing framework [1].”

Please name the framework

- “The seven existing domains of AEs were confirmed: cognitive, perceptual, affective, somatic, conative, sense of self, and social.”

Take out “the”, and I suggest changing language from “confirmed” to “observed”, see below.

**Intro**

- “Mindfulness-Based Interventions (MBIs) are widely used in both clinical and non-clinical populations, and although the benefits of MBIs in psychiatric populations are well documented [4], the same applies to MBIs.”

Grammatically confusing. What is “the same” that applies to MBIs?

- As mentioned earlier, Britton, et al. 2021 should be cited, as that article outlines a methodological approach and has described base rates for AEs. Also Goldberg, et al. 2021 will be relevant.
- I suggest caution in generalizing Lindahl, et al.’s study to MBIs, as that research was done with long-term Buddhist meditators. This article mentions this as an aside at the end, but it should be clear from the outset.

**Method**

- The authors mention that this research relied on an adapted version of MBCT. Reasons and implications of adaptation should be stated (perhaps in discussion is OK).
- Weekly completion of questionnaires is a definite strength. However, if only 12 AEs that were a-priori deemed most relevant to BD were indicated, this may also leave important AEs out. AEs may have as much to do with the intervention as with the population, so making the items only focused on the specifics of the population may miss important characteristics. By way of analogy, an (S)AE of aspirin is Reye’s syndrome. However, if probing AEs of aspirin among low back pain sufferers, only asking about low back-relevant AEs would miss this serious AE. To be clear, the current questions seem appropriate and—if selecting 12—the items used appear to be good choices even if they do leave out (for example) change in social relationships.
- Small concern: IH knew the patients and was responsible for recruiting them. Was there any concern that patients may under-report AEs to the person who recruited them, especially if she is a junior researcher?
- The authors discuss the construal of AEs as ultimately positive in many cases. For reference in the varied interpretation of meditation-related AEs, and some of the context around positive vs. negative interpretation of AEs as “progress”-related, they may also find a recent paper by Lindahl, et al. (2020) relevant.
- Was any kind of intent to treat approach used with drop-outs? Was there any indication of why people dropped out and whether this may have been related to AEs? Also, it’s not clear to what extent drop-outs are taken into account in the current analyses. A problem sentence is: “Of these, 29 (50%) patients reported one or more AEs and were invited for a semi-structured interview…”

“of these” is confusing, since prior to this sentence a general sample is listed followed by number drop-outs. So is this referring to all participants prior to drop-out, or after excluding drop-outs?

- The description of qualitative analyses is quite sparse. Are there any data on initial rates of agreement between coders? What was the “existing framework” for directed content analysis? Etc.
- Was chronicity/duration of AEs monitored? If so, how? The authors indicate later in the paper that enduring AEs were not observed, but it is not clear how they are able to make such a statement, especially if AEs were not monitored after end of trial.

**Results**

- “In the current study, 41 of the 59 categories of the phenomenology codebook by Lindahl et al. [1] were confirmed (Supplementary Table 3).”

I suggest stating “observed” instead of “confirmed”, since the design of the study is not well suited to confirm or disconfirm these categories (i.e., the authors did not produce an independent coding system and find it to accord with the Lindahl, et al. themes, nor did they sample an adequate number of participants (22 vs. approximately 60 in the Lindahl et al. study). To be clear this is not a problem with the study or its methods, which are sound. Rather, this is just about what the study is and isn’t positioned to do. I therefore suggest “observed.”

- “Both the number of patients who reported AEs and the average number of different AEs per patient declined over the course of MBCT, from 12 (21%) in week 1 to 8 (14%) in week 7 and from 157 3.6 in week 1 to and 1.5 in week 7, respectively (see Fig 3a and Fig 3b).”

This is a bit confusing – are these total patients or averaged numbers of AEs? I am assuming that it’s patients followed by averages, but please make this clear.

- “Changes in doubt, faith, trust, or commitment were frequently reported as well, which referred to insecurities about patients’ ability to learn mindfulness skills and retain a stable a mood, and doubts about 172 whether mindfulness could be harmful in the light of the occurring AEs.”

What is “frequently”?

- “As no SAEs were found in this study, the text below will only mention AEs.”
- The authors do not explain how “seriousness” was determined, a very important omission to correct. This explanation would naturally fall in the “monitoring serious adverse effects” section, but that section only includes the questionnaire and interview approach.
- Please define each of the 4 new categories for influencing factors. I.e., the authors write “Patients mentioned several factors that existed before the start of MBCT and that might predispose to the occurrence of AEs.” Are these factors deemed by the patients, or by the researchers, to be predisposing? And if by the researchers, based on what criteria? How were these predisposing factors (i.e., personality traits) identified – were they spontaneously reported by participants, or queried by interviewers?
- Please explain the categorization of “trying to continue the meditation exercises despite the occurrence of AEs” as a maladaptive (automatic) coping strategy, given that instructions to “meditate through it” are a conspicuous concern in popular meditation instruction. If this was in line with existing instruction and literature on meditation, calling it “automatic” localizes this issue to the patient, rather than placing appropriate responsibility with instructors (perhaps not those in the present study) who repeat this instruction.
- “Mindfulness skills” as a mitigating factor is not an immediately clear category – please provide a definition/explication, including how the category was generated. Were these described as “mindfulness skills” by participants, or categorized as such by the researchers? How are these different from “ground activities” (which include focusing on the breath) for instance?
- It will be important to resolve the following concern: The article initially states that most patients interpreted the AEs to be ultimately therapeutic or positive. However, the *Consequences* section begins by writing “First of all, some patients believed that the AEs would persist or even worsen over time and tended to avoid practicing mindfulness altogether in order to prevent this.” Or at the end of that paragraph, “Some patients experienced AEs as merely having negative impact, because they did not feel that they had learned anything from it.” Given the small number of participants, this is non-trivial and would seem to indicate that the earlier statement be revised. One possible revision would be to add something like “although some only experienced AEs as negative.” With monitoring adverse effects, it is important not to elide the experiences of those patients. It is also important to bear in mind that the full extent of distress may not be observed within the time window of the study. For example, when comparing AEs that did vs. did not reduce over time (Supplementary Table 4), **the number that do not decrease outnumber those that did.** Likewise, the “majority” who interpreted the AEs as part of a growth process was 11 vs. 8 who did not, which I is a VERY SLIM majority. Since an AE like depersonalization can seem fine within the time frame of an 8-week MBCT treatment but, without improvement, can become highly distressing after 3 months, it is inappropriate to characterize these as mostly positive overall.
- The *Consequences* section again mentions specific “mindfulness skills” which makes me wonder whether these were queried in the interview, or whether they were ultimately categorized as such by the researchers.
- Given the spread of those who did and did not find AEs to ultimately be beneficial, it might be useful to see the numbers behind these differences.
- “During the course of MBCT patients gradually learn to develop acceptance and self-kindness, which have been shown to reduce psychological distress [10, 15, 16]. This might explain why the number of AEs reported by patients starts to decline after three weeks. These results suggest that allowing difficult or adverse emotions can result in a reduction of associated fears and habituation. This is in accordance with exposure-based therapies, where a temporary increase of anxiety is considered a necessary vehicle to change patients’ beliefs and emotions [7]” This may be overstating the case, and I would suggest caution in making such an interpretation given the kind of—and amount of—data here. For instance, an alternative explanation is that, as with many adverse experiences, impacts are shorter-term, likelier to occur in tandem with any novel experience, and that they recover on their own because people are by and large resilient. Decline in reporting could be due to Hawthorne effects, as patients who report an AE 3 weeks in a row may be reluctant/tired/habituated to it on week 4 even though they still experience it. It is thus a strength of the study that these patients were subsequently interviewed (and yet the interviews did not suggest that decrease was the majority experience). Finally, research suggests that early losses in treatment are not good predictors of longer-term treatment gains (e.g. “it will get worse before it gets better”)—this is a common assumption but is not well supported (Flückiger et al., 2013; Koffmann, 2018, 2020; Lutz et al., 2013).
- “In the current study, with a vulnerable population of patients with BD, MBCT seemed reasonably safe.”

This is a value judgment. See paper by Britton, et al. (2021) for discussing safety.

- Given that one patient died by suicide (a low base rate event), I am concerned about the authors’ justification in saying they did not observe suicidality as an AE among patients.
- Given the low base rates of harms in general, it is not justified based on lack of observation in this trial to say that these do not occur.
- “Both precipitating and perpetuating factors provide insight into ways to improve MBIs, for example by managing expectations and inform patients about the possibility that symptoms may initially get worse” See earlier comment that “things get worse before they get better” is not well borne out in psychotherapy, and that early losses do not predict later gains.
- “Informing patients in advance may prevent them from dropping out due to these AEs.” Why is it inappropriate to drop out due to AEs? This seems like it would be a reasonable strategy with many other therapies. It also stands in contrast with the researchers’ observations that persisting with the practice is a potential exacerbating factor. I suggest changing this language to indicate that patients should received informed guidance about their options to drop out, switch therapies, etc. in the event of AEs.
- The authors state that long-term AEs were not observed, but since all AEs did not disappear at end of study, how can they know?
- Not sure why the patterns described in strengths and limitations are deemed “over”-reporting. It seems appropriate to query AEs and have a system for determining whether these were harmful, but the methods are well designed to correct for any over-estimation due to the sensitivity of the instruments.
- A limitation is that drops due to AEs were not recorded.

References

Britton, W. B., Lindahl, J. R., Cooper, D. J., Canby, N. K., & Palitsky, R. (2021). Defining and Measuring Meditation-Related Adverse Effects in Mindfulness-Based Programs. *Clinical Psychological Science*, 2167702621996340. https://doi.org/10.1177/2167702621996340

Flückiger, C., Holtforth, M. G., Znoj, H. J., Caspar, F., & Wampold, B. E. (2013). Is the relation between early post-session reports and treatment outcome an epiphenomenon of intake distress and early response? A multi-predictor analysis in outpatient psychotherapy. *Psychotherapy Research*, *23*(1), 1–13. https://doi.org/10.1080/10503307.2012.693773

Goldberg, S. B., Lam, S. U., Britton, W. B., & Davidson, R. J. (2021). Prevalence of meditation-related adverse effects in a population-based sample in the United States. *Psychotherapy Research: Journal of the Society for Psychotherapy Research*, 1–15. https://doi.org/10.1080/10503307.2021.1933646

Koffmann, A. (2018). Has growth mixture modeling improved our understanding of how early change predicts psychotherapy outcome? *Psychotherapy Research*, *28*(6), 829–841. https://doi.org/10.1080/10503307.2017.1294771

Koffmann, A. (2020). Early trajectory features and the course of psychotherapy. *Psychotherapy Research*, *30*(1), 1–12. https://doi.org/10.1080/10503307.2018.1506950

Lindahl, J. R., Cooper, D. J., Fisher, N. E., Kirmayer, L. J., & Britton, W. B. (2020). Progress or Pathology? Differential Diagnosis and Intervention Criteria for Meditation-Related Challenges: Perspectives From Buddhist Meditation Teachers and Practitioners. *Frontiers in Psychology*, *0*. https://doi.org/10.3389/fpsyg.2020.01905

Lutz, W., Ehrlich, T., Rubel, J., Hallwachs, N., Röttger, M.-A., Jorasz, C., Mocanu, S., Vocks, S., Schulte, D., & Tschitsaz-Stucki, A. (2013). The ups and downs of psychotherapy: Sudden gains and sudden losses identified with session reports. *Psychotherapy Research*, *23*(1), 14–24. https://doi.org/10.1080/10503307.2012.693837

Schlosser, M., Sparby, T., Vörös, S., Jones, R., & Marchant, N. L. (2019). Unpleasant meditation-related experiences in regular meditators: Prevalence, predictors, and conceptual considerations. *PLOS ONE*, *14*(5), e0216643. https://doi.org/10.1371/journal.pone.0216643
