# Supplementary material for: Adverse or therapeutic? A mixed-methods study investigating adverse effects of Mindfulness-Based Cognitive Therapy in bipolar disorder
Source: PLoS One. 2021 Nov 4;16(11):e0259167. doi: 10.1371/journal.pone.0259167 (PMC8568103; doi:10.1371/journal.pone.0259167)
Supplement: Attachment — Submitted filename: Response to Reviewers.docx [file pone.0259167.s009.docx]

**Reviewer #1: I am generally happy with the authors' responses to my previous few comments. However, I still have issues with the methods section. I did mention a logistic regression for example before and the authors responded that this is presented in the results section. Perhaps i should have rephrased my point original point, but, from my point of view, if it's not described in the methods section, it did not happen. And the analysis plan for the quants is a single sentence that does not mention such an analysis. So I would urge the authors to expand the data analysis section to include all the analyses they conducted, clearly.**
[Authors] We thank the reviewer for the attentiveness regarding the methodology of this article, as it is important that this is as transparent as possible.

The previous comment the reviewer refers to is as follows: “*Characteristics associated with SAEs are not examined, in say a multiple logistic regression (or even a zero-inflated Poisson regression to capture multiple SAEs).”*

The authors responded with the following: *“We did explore whether baseline characteristics (e.g. age, gender, co-morbid psychiatric disorders, number of previous mood episodes, duration of illness, baseline depression, etc.) differed between patients who did and did not report (S)AEs. We did not find any differences, except for baseline anxiety (see page 7, line 178). This is an important finding, and we elaborated on this in the discussions section (page 14, line 402-407).”*

We want to apologize for not addressing the reviewers’ question sufficiently. We did not conduct a multiple logistic regression to investigate which characteristics are associated with SAEs. We did however explore if there were any differences between the two groups (did or did not experience AEs) regarding these characteristics by means of chi-square and independent t-tests, as mentioned in the methods section (page 6, lines 147-150): *“Quantitative data were analysed using SPSS version 25.0 [15], conducting chi-square and independent t-test statistics to compare demographic and clinical variables between patients who did and did not report (S)AEs.”* As there was only one difference between those groups (baseline anxiety), we think that conducting a multiple logistics regression analysis will not be of added value. Moreover, our sample population might be too small for making a multivariate model. We hope the reviewer agrees with our decision to only look for differences between groups by means of chi-square and independent t-test statistics, and not by conducting a multivariate logistic regression.

**Reviewer #2: I would like to commend the improvements and serious consideration that the authors have given to this article and its revision. I am supportive of the changes that have been made overall, and would like to recommend acceptance.**
[Authors] We thank the reviewer for the helpful comments which helped us to improve our manuscript.
